# *N*-methyl-*N*-nitrosourea-induced retinal degeneration in mice is independent of the *p53* gene

**Published:** 2009-12-30

**Authors:** Katsuhiko Yoshizawa, Maki Kuwata, Ayako Kawanaka, Norihisa Uehara, Takashi Yuri, Airo Tsubura

**Affiliations:** Second Department of Pathology, Kansai Medical University, Moriguchi, Osaka, Japan

## Abstract

**Purpose:**

A single systemic administration of *N*-methyl-*N*-nitrosourea (MNU) causes retinal degeneration involving photoreceptor cell loss within 7 days. MNU-induced photoreceptor cell loss is due to apoptosis and is a reliable animal model for human retinitis pigmentosa. The purpose of this study was to determine if *p53* contributes to the development of MNU-induced retinal degeneration in mice.

**Methods:**

Eight-week-old *p53^−/−^*, *p53^+/−^*, and *p53^+/+^* mice received an intraperitoneal injection of 60 mg/kg bodyweight of MNU. Age-matched *p53^+/+^* mice received the vehicle only (physiologic saline containing 0.05% acetic acid). Mice were sacrificed and necropsied 7 days after the treatment. Both eyes were examined histologically and morphometrically to determine retinal thickness, photoreceptor cell ratio, and retinal damage ratio.

**Results:**

No mice died during the experiment, but the *p53* null mice treated with MNU had a statistically significant weight loss compared to the other groups. Histologically, all MNU-treated mice, regardless of *p53* gene status, experienced retinal degeneration characterized by photoreceptor cell loss (the disappearance of the outer nuclear layer and photoreceptor layer) in both the central and peripheral retina. All MNU-treated mice had significantly decreased retinal thickness and photoreceptor cell ratios at the central and peripheral retina and an increased retinal damage ratio compared to the vehicle-treated control. The retinal changes caused by MNU in *p53^+/+^*, *p53^+/−^*, and *p53^−/−^* mice were not significantly different and hence were related to a *p53*-independent apoptotic mechanism.

**Conclusions:**

Because the absence of *p53* did not prevent photoreceptor cell loss, we conclude that *p53* is not essential for MNU-mediated photoreceptor cell degeneration.

## Introduction

Retinitis pigmentosa (RP) is characterized by early nyctalopia and noninflammatory, bilateral, progressive, degenerative pigmentary retinopathy; the photoreceptor loss is followed by perivascular pigment deposition within the retina [1,2]. RP is a heterogeneous group of inherited retinal disorders, and more than 160 different mutations of genes encoding proteins with remarkably diverse functions are known to cause photoreceptor degeneration (RetNet). Animal models of retinal degeneration are used to elucidate the mechanism of human RP [3,4] and to search for a treatment or a cure [2,5]. Mutant mice used as models for RP [6] include mice carrying the rodless retina or retinal degeneration (*rd)* gene or the retinal degeneration slow *(rds*) gene; these mice experience photoreceptor cell death caused by apoptosis [7,8]. In addition to inherited RP models, there are chemically induced retinal degeneration models. Mammalian eyes are highly sensitive to toxic substances, and *N*-methyl-*N*-nitrosourea (MNU), an alkylating agent that targets photoreceptor cells, rapidly induces retinal damage via apoptosis in animal species, including the mouse [5,9]. Within 7 days after MNU exposure, active signs of photoreceptor degeneration are indistinct due to photoreceptor cell loss, and the inner nuclear layer is either in direct contact with the choroid or is separated from it by a few layers of cells [10].

p53 is a transcription factor that regulates the activity of genes involved in cell-cycle arrest, apoptosis, anti-angiogenesis, differentiation, DNA repair, and genomic stability [11,12]. p53 mediates apoptosis in response to DNA damage and cell-cycle perturbations; however, various forms of p53-independent apoptosis have also been identified [13,14]. Apoptosis occurs widely during the development of nerve systems [15,16], including the visual system [17]. Developmental apoptosis in the nervous system is generally *p53* independent since most *p53*-deficient mice develop normally [13,18]. However, retinal ganglion cells provide a convenient model system to investigate *p53*-dependent apoptosis during development [17].

Our goal in the present study is to elucidate the effect of *p53* on MNU-induced photoreceptor degeneration in mice. We also discuss the involvement of *p53* in different types of retinal cells and on photoreceptor cell loss caused by different stimuli.

## Methods

### Animals

Female B6;129-*Trp*53^tm1Brd^ N4 (*p53^−/−^*), N5 (*p53^+/−^*), and N5 (*p53^+/+^*) mice were purchased at 5 to 8 weeks of age from Taconic (Germantown, NY). *p53* knockout and wild-type mice were generated in 129/Sv-derived embryonic stem cells (AB1) and were backcrossed onto a C57BL background [19]. In brief, resultant chimeras were backcrossed to C57BL/6J for two generations (N2). Then, the mice were backcrossed to N3 for caesarean derivation and backcrossed to N4 immediately after derivation. The homozygous colony was maintained at N4 through the mating of heterozygous females with homozygous males. The heterozygous colony was maintained at N5 through the mating of N4 male homozygotes to C57BL/6NTac females. The wild-type control colony was maintained at N5 through the mating of N4 wild-type mice to C57BL/6NTac mice. There have been no reports of spontaneous retinal degeneration in these knockout and wild-type mouse colonies. Each animal’s genotype was determined by the supplier before shipment. Mice were maintained in specific pathogen-free conditions and had free access to a commercial mix-feed diet (CMF, 30 kGy; Oriental Yeast, Chiba, Japan) and water. Animals were housed in plastic cages with paper-chip bedding (Paper Clean; SLC, Hamamatsu, Japan) in an air-conditioned room at 22±2 °C and 60%±10% relative humidity with a 12 h:12 h light–dark cycle. All cages were placed in the same row, and the illumination intensity in the cages was less than 60 lux. All procedures were in accordance with the guidelines for animal experimentation at Kansai Medical University, Moriguchi, Japan.

### Chemical and dose formulation

MNU (Sigma; St. Louis, MO) was kept at −80 °C in the dark. The MNU solution was dissolved in physiologic saline containing 0.05% acetic acid just before use. Mice received one intraperitoneal (i.p.) injection of MNU at a dose of 60 mg/kg or an injection of vehicle only (physiologic saline containing 0.05% acetic acid) [10].

### Experimental procedures

At 8 weeks of age, three *p53^−/−^*, seven *p53^+/−^*, and seven *p53^+/+^* mice received an i.p. injection of MNU (60 mg/kg bodyweight), and seven *p53^+/+^* mice received an i.p. injection of vehicle. All mice were observed daily for clinical signs of toxicity and were weighed at the time of MNU injection and on the day of sacrifice. All mice were inhalated with isoflurane (Forane; Abbot Japan, Tokyo, Japan) and sacrificed 7 days after MNU or vehicle treatment. At the time of sacrifice, both eyes were quickly removed and complete necropsies were conducted on all animals.

### Tissue fixation and processing

Eyes from each mouse were fixed overnight in methacarn (60% methanol, 30% chloroform and 10% acetic acid) [20] and embedded in paraffin; then, 4-μm-thick sections were prepared and stained with hematoxylin and eosin (H&E). Eye sections were cut along a line parallel to the optic axis and nerve (including the ora serrata). H&E sections of the retina were scanned to create digital images with a high-resolution digital slide scanner (NanoZoomer 2.0 Digital Pathology, Hamamatsu Photonics, Hamamatsu, Japan). The NanoZoomer-Digital-Pathology-Annotations (ndpa)-image files were opened in color mode by specific viewer software, NDP.view (Hamamatsu Photonics).

### Morphometric analysis of retinal thickness, photoreceptor cell ratio, and retinal damage ratio

As described previously [20,21], we used NDP.view to measure the total retinal thickness (from the internal limiting membrane to the pigment epithelium), inner retinal thickness (from the internal limiting membrane to the outer plexiform layer), and outer retinal thickness (from the outer nuclear layer to the pigment epithelial cell layer). The measurements were collected at the central retina (approximately 400 μm from the optic nerve) and the peripheral retina (approximately 400 μm from both sides of the ciliary bodies). The photoreceptor cell ratio was calculated as [(outer retinal thickness)/(total retinal thickness)]×100. To determine the area of retinal damage, the entire length of the retina and the length of the damaged area in H&E preparations were measured. A damaged retina was designated as the presence of less than four rows of photoreceptor nuclei in the outer nuclear layer [20,21], and the retinal damage ratio was calculated as [(length of damaged retina/whole retinal length)]×100. Two toxicology pathologists (K.Y. and A.T.) certified by the Japanese Society of Toxicologic Pathology performed the histopathological and morphometrical evaluations, according to previously described histopathological terminology and diagnostic criteria [20,21].

### Statistical analysis

All discrete values, expressed as the mean±standard error (SEM), were analyzed with the two-tailed independent Student *t*-test for unpaired samples after confirming the homogeneity of variances. MNU-treated retinas (regardless of p53 status) were compared with vehicle-treated p53^+/+^ retinas, and the MNU-treated retinas were compared based on their p53 status. A p value <0.05 was considered to show a statistically significant difference.

## Results

### General remarks

**Figure 1:**
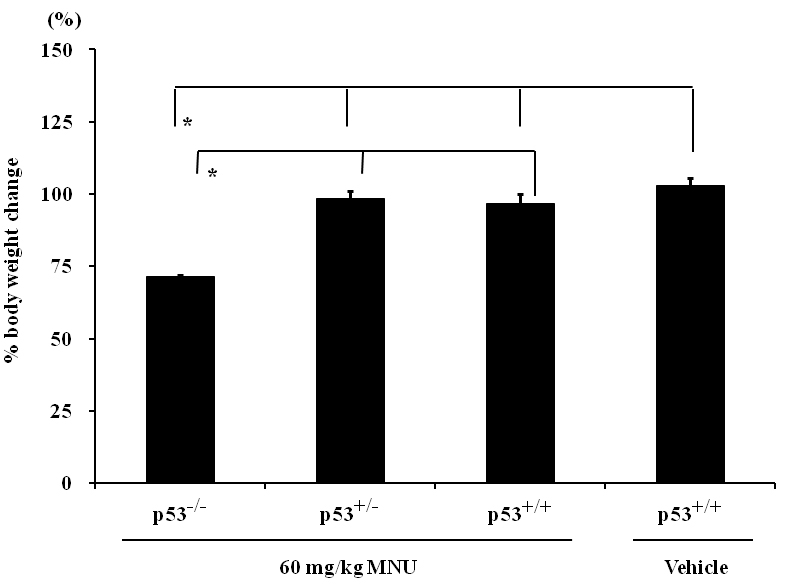
Percent bodyweight change of initial bodyweight in N-methyl-N-nitrosourea (MNU) -treated *p53^−/−^*, *p53^+/−^*, and *p53^+/+^* mice and vehicle-treated *p53^+/+^* control mice 7 days after the treatment. The MNU-treated *p53* null mice experienced a significant decrease in bodyweight as compared to vehicle-treated and MNU-treated *p53^+/−^* and *p53^+/+^* mice. Asterisk is p<0.01 and compared with three other organs.

All mice remained healthy during the 7-day experimental period, and the MNU-treated *p53^+/−^* and *p53^+/+^* MNU did not experience weight loss (Figure 1). However, the MNU-treated *p53^−/−^* mice revealed a statistically significant weight loss; their bodyweight 7 days after treatment was 70.3% of their initial weight (Figure 1). In the MNU-treated *p53^−/−^* mice, a severe degree of atrophic changes was detected in the lymph–hematopoietic system (thymus, spleen, lymph node, and bone marrow).

### Retinal change caused by *N*-methyl-*N*-nitrosourea

**Figure 2:**
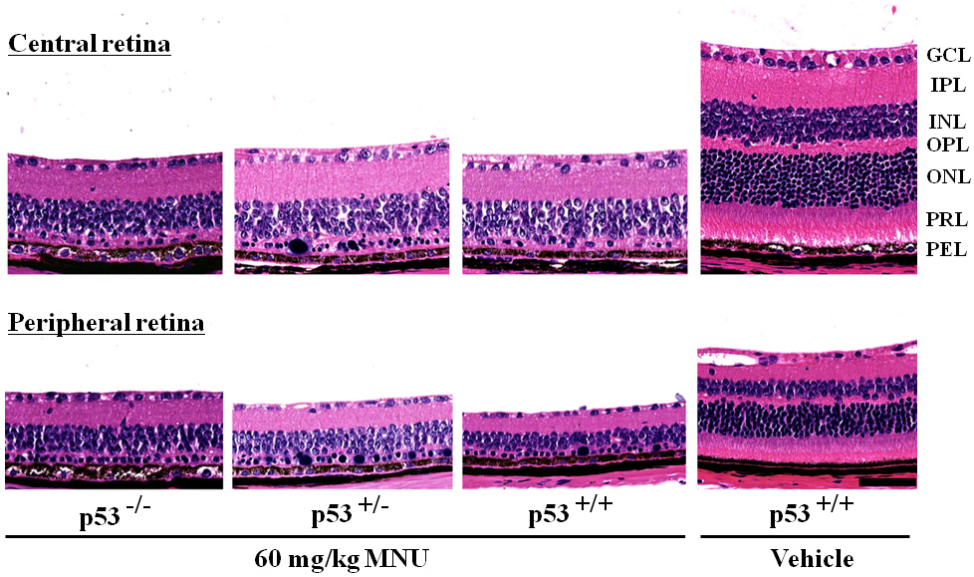
Retinal changes 7 days after a single systemic administration of 60 mg/kg N-methyl-N-nitrosourea (MNU) to *p53^−/−^*, *p53^+/−^*, and *p53^+/+^* mice. In MNU-treated *p53^−/−^*, *p53^+/−^*, and *p53^+/+^* mice, the outer nuclear layer and photoreceptor layer disappeared in both the peripheral and central retina. Equivalent levels of severe retinal degeneration were detected in MNU-treated *p53^−/−^*, *p53^+/−^*, and *p53^+/+^* mice. GCL, ganglion cell layer; IPL, inner plexiform layer; INL, inner nuclear layer; OPL, outer plexiform layer; ONL, outer nuclear layer; PRL, photoreceptor cell layer; and PEL, pigment epithelial cell layer. The sections were stained with hematoxylin and eosin. The scale bar represents 70 μm.

Retinal histology was studied 7 days after MNU treatment. In vehicle-treated *p53^+/+^* mice, photoreceptor nuclei at the central retina consisted of more than ten layers of cells, and the peripheral retina consisted of more than seven layers of cells (Figure 2). In contrast, regardless of *p53* status, the outer nuclear layer of MNU-treated mice contained no photoreceptor nuclei or only a few layers of nuclei at both the central and peripheral retina (Figure 2). The remaining photoreceptor nuclei in MNU-treated mice were densely stained; their chromatin was clumped, and large basophilic bodies were present in between the remaining photoreceptor nuclei. The MNU-induced changes were restricted to photoreceptor cells.

### Morphometric analysis of retinal damage

**Figure 3:**
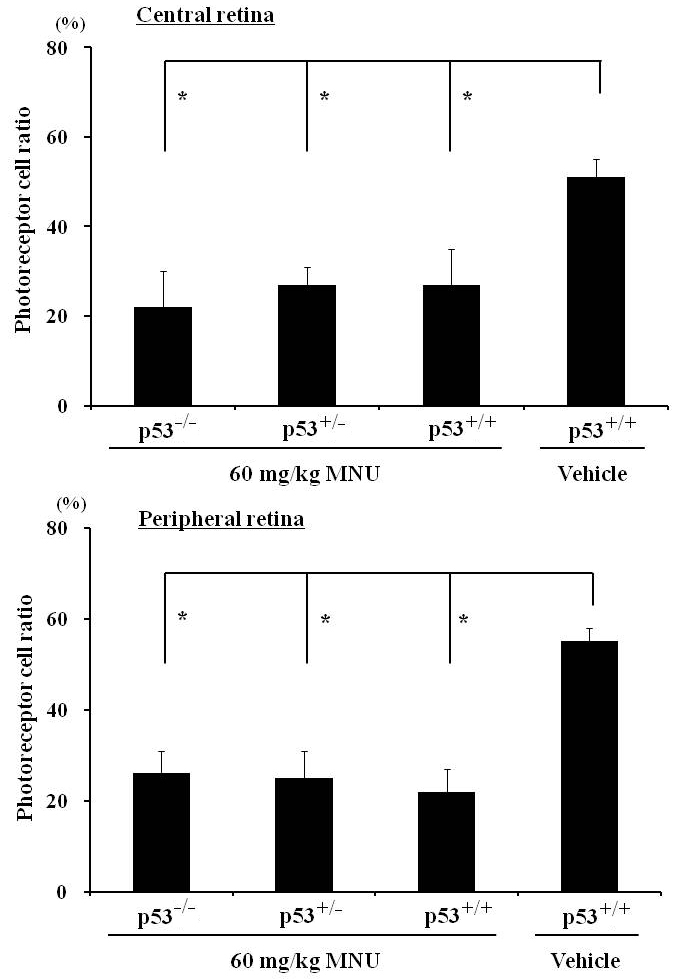
Photoreceptor cell ratio in the central and peripheral retina 7 days after a single systemic administration of 60 mg/kg N-methyl-N-nitrosourea (MNU) to *p53^−/−^*, *p53^+/−^*, and *p53^+/+^* mice. Photoreceptor cell ratio was calculated as [(outer retinal thickness)/(total retinal thickness)]×100. In mice treated with MNU, regardless of their *p53* status, the total retinal thickness and the outer retinal thickness of the central and peripheral retina were significantly decreased, resulting in a statistically significant decrease in the photoreceptor ratio of MNU-treated mice compared to vehicle-treated controls. Asterisk is p<0.01 and compared with vehicle controls.

Seven days after MNU treatment, the total retinal thickness and outer retinal thickness of all mice, regardless of *p53* status, was significantly decreased in both the central and peripheral retina compared to the vehicle-treated *p53^+/+^* mice. In the MNU-treated mice, neither the total retinal thickness nor the outer retinal thickness at the central and peripheral retina was significantly different based on the *p53* status. However, MNU did not cause any changes in inner retinal thickness as the inner retinal thickness in MNU-treated mice was comparable to that of vehicle-treated mice (data not shown). To further evaluate the effects of *p53* on retinal thickness, the photoreceptor cell ratio was calculated (Figure 3). In vehicle-treated *p53^+/+^* mice, the outer retinal ratio at the central and peripheral retina was 51±4% and 55±3%, respectively, while in MNU-treated *p53^−/−^*, *p53^+/−^*, and *p53^+/+^* retina, it decreased to 22±8% and 26±5%, 27±4% and 25±6%, and 27±8% and 22±5%, respectively. Regardless of the *p53* gene status, the photoreceptor cell ratio in the central and peripheral retina was not significantly different in the three groups of MNU-treated mice.

**Figure 4:**
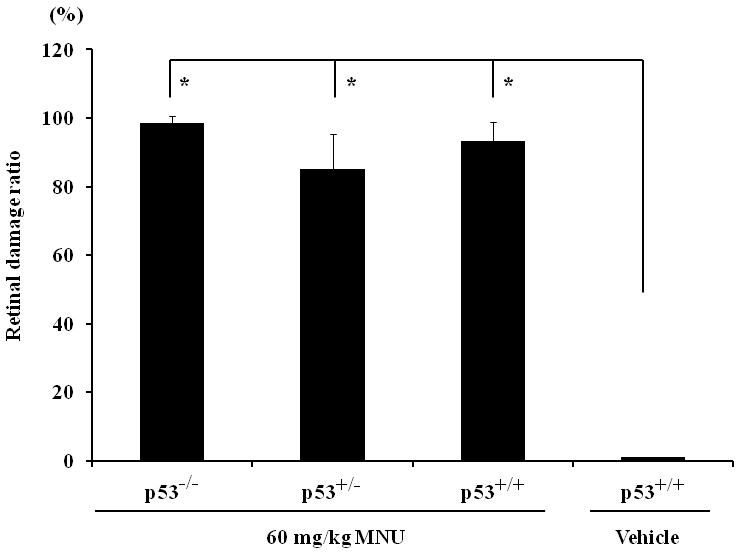
Retinal damage ratio in N-methyl-N-nitrosourea (MNU)-treated *p53^−/−^*, *p53^+/−^*, and *p53^+/+^* mice. The retinal damage ratio was calculated as the percentage of length of retina composed of less than four photoreceptor cells on the length of whole retina. Asterisk is p<0.01 and compared with vehicle controls.

To evaluate the degree of disease progression, the retinal damage ratio was compared among the groups (Figure 4). In MNU-treated *p53^−/−^*, *p53^+/−^*, and *p53^+/+^* retina, the retinal damage ratio was 98.5±2.1%, 85.0±10.2%, and 93.3±5.6%, respectively. The MNU-induced damage reached almost the entire retina, and the *p53* gene status did not influence the disease progression. As anticipated, the retinal damage ratio in vehicle-treated *p53^+/+^* mice was 0%.

## Discussion

*p53* plays an important role in normal eye development. In *p53^−/−^* mice derived from the Jackson Laboratory colony, the hyaloid vasculature persists and develops into a fibrovascular retrolental plaque that leads to cataract formation in young adult mice [22,23]. This developmental ocular anomaly occurs in *p53*-deficient C57BL/6 mice but not in the *p53*-deficient 129/Sv strain, indicating the importance of background strains [23]. The *p53*-deficient mice used in the present study have no such abnormalities.

*p53* is a major regulator of cell death in response to various stresses that involve DNA damage [24]. However, both *p53*-dependent and *p53*-independent mechanisms may exist in the retinal degeneration models depending on the type and degree of stress to the retina [25,26]. MNU causes DNA adduct formation in photoreceptor nuclei, followed by increased poly(ADP-ribose) polymerase activity. It leads through inactivation of nuclear factor-κB and the activation of Jun N-terminal kinase/activator protein. Then it causes downregulation of Bcl-2, upregulation of Bax, and the activation of Caspase-3, -6, and -8 [5,20,27]. In the present study, retinal degeneration that was characterized by the disappearance of the outer nuclear layer and the photoreceptor layer in both the peripheral and central retina was detected in *p53^−/−^* mice, *p53^+/−^* mice, and *p53^+/+^* mice 7 days after a single i.p. injection of 60 mg/kg MNU. Morphometric indices, such as the retinal photoreceptor ratios in the central and the peripheral retina and the retinal damage ratio, revealed that the degree of retinal lesions in MNU-treated *p53^−/−^* mice was similar to that of MNU-treated *p53^+/−^* and *p53^+/+^* mice. Our present results indicate that MNU induces *p53*-independent retinal degeneration. The MNU-treated *p53^−/−^* mice revealed a statistically significant weight loss compared to the other groups. Histopathologically, a severe degree of atrophic changes was detected in the lymph–hematopoietic system (thymus, spleen, lymph node, bone marrow) in MNU-treated *p53^−/−^* mice. The cause of the significant weight loss in MNU-treated *p53^−/−^* mice seems to be due to systemic toxicity induced by MNU.

Calpains (calcium-dependent cysteine proteases) are activated by increased cellular Ca^2+^ concentration. Excitotoxic stimuli results in a massive Ca^2+^ influx into the target cells, and poly(ADP-ribose) polymerase activation caused by DNA damage further dysregulates Ca^2+^, which results in calpain activation, and is followed by cell death. Total Ca^2+^ in the retina of MNU-treated rats is significantly increased and calpain activity is dramatically increased 1 and 3 days after MNU and decreased at day 7 [28,29]. The role of calpain family proteases in the *p53*-independent apoptosis pathways after MNU exposure is unclear and needs further investigation.

Light-induced photoreceptor cell death is *p53* independent. When *p53^−/−^* and *p53^+/+^* mice were dark adapted for 36 h and then exposed to more than 8,000 lux for 2 h, the degree of photoreceptor cell damage was the same in both genotypes [30,31]. In an inherited model of retinal degeneration, mice carrying the *rd* gene develop photoreceptor degeneration early in life. Although the retinal development in *rd* mice is comparable to that in normal mice at 8 days of age, the number of photoreceptor in *rd* mice is reduced by 11 days of age and the photoreceptor cells are completely missing or reduced to a single layer of cells by 20 days of age [32]. When the retinas of 10- to 20-day-old *p53^−/−^* *rd/rd* and *p53^+/+^* *rd/rd* mice were compared, the photoreceptor cell loss was indistinguishable, suggesting that photoreceptor apoptosis in *rd* mice occurs in a p53-independent manner [33,34]. In mice carrying the *rds* gene, photoreceptor cell loss starts at 2 weeks of age and progresses slowly with complete loss occurring 1 year after birth [3]. A comparison of *p53^−/−^* *rds/rds* mice and *p53^+/+^* *rds/rds* mice revealed that the retina develop similarly [35]. Although a slight delay in photoreceptor apoptosis occurred between 16 and 26 days of age in *p53^−/−^* *rds/rds* mice, the amount of photoreceptor cell loss was similar at 35 days of age, indicating that photoreceptor apoptosis in *rds* mice is p53 independent. Thus, inherited as well as excitotoxic stimuli cause *p53*-independent apoptosis in photoreceptor cells.

p53 may regulate apoptosis differently in different types of retinal cells. Retinal ganglion cell (RGC) death is induced by N-methyl-D-aspartate (NMDA) [36]. When 160 nM NMDA was intravitreally injected into *p53^+/+^*, *p53^+/−^*, and *p53^−/−^* mice, the *p53^+/+^* and *p53^−/−^* mice exhibited a statistically equivalent amount of RGC loss at 4 days after the treatment, while the *p53^+/−^* mice had significantly attenuated cell loss. In *p53^+/+^*, *p53^+/−^*, and *p53^−/−^* mice, the cleavage of poly(ADP-ribose), which is a substrate for caspases, was found in *p53^+/+^* and *p53^+/−^* eyes but not in *p53^−/−^* eyes. Although the mechanism that protects *p53^+/−^* mice from RGC death needs further study, the NMDA-induced RGC death was *p53* dependent; however, *p53*-independent pathways also exist.

Radiation-induced apoptosis in retinal progenitor cells in the inner nuclear layer is *p53* dependent [37,38]. When 5-day-old *p53^+/+^* and *p53^−/−^* mice received 14 Gy whole body irradiation [38], the *p53^+/+^* retina contained activated p53 in the retinal progenitor cells of the inner retina (but not in the outer retina) 2 h after irradiation, and apoptotic cells appeared in the inner nuclear layer 5 h after irradiation; however, retinal cell death was completely abrogated in the *p53^−/−^* retina. In 3- to 4-day-old *p53^+/+^*, *p53^+/−^*, and *p53^−/−^* mice, the *p53^−/−^* retina was completely resistant to 14 Gy irradiation, while a *p53* gene dosage effect was observed after 2 Gy irradiation; *p53^−/−^* retinas were completely protected, while *p53^+/−^* retinas were partially protected [37]. Thus, the involvement of *p53* in radiation-induced retinal cell death seems to be cell-type specific and dose dependent.

In conclusion, after a single i.p. injection of 60 mg/kg MNU, retinal degeneration occurred in *p53^−/−^* and *p53^+/−^* mice. The degree of retinal degeneration in these mice was similar to that of MNU-treated *p53^+/+^* mice. Therefore, *p53* may not be essential for MNU-induced photoreceptor degeneration in mice. Detailed investigations of *p53* gene function in human RP are needed for a better understanding of the pathogenesis of RP.
